# The Evolution of Extracellular Fibrillins and Their Functional Domains

**DOI:** 10.1371/journal.pone.0033560

**Published:** 2012-03-16

**Authors:** Adam Piha-Gossack, Wayne Sossin, Dieter P. Reinhardt

**Affiliations:** 1 Department of Anatomy and Cell Biology, McGill University, Montreal, Quebec, Canada; 2 Department of Neurology and Neurosurgery, McGill University, Montreal, Quebec, Canada; 3 Faculty of Dentistry, McGill University, Montreal, Quebec, Canada; Hospital for Sick Children, Canada

## Abstract

Fibrillins constitute the major backbone of multifunctional microfibrils in elastic and non-elastic extracellular matrices, and are known to interact with several binding partners including tropoelastin and integrins. Here, we study the evolution of fibrillin proteins. Following sequence collection from 39 organisms representative of the major evolutionary groups, molecular evolutionary genetics and phylogeny inference software were used to generate a series of evolutionary trees using distance-based and maximum likelihood methods. The resulting trees support the concept of gene duplication as a means of generating the three vertebrate fibrillins. Beginning with a single fibrillin sequence found in invertebrates and jawless fish, a gene duplication event, which coincides with the appearance of elastin, led to the creation of two genes. One of the genes significantly evolved to become the gene for present-day fibrillin-1, while the other underwent evolutionary changes, including a second duplication, to produce present-day fibrillin-2 and fibrillin-3. Detailed analysis of several sequences and domains within the fibrillins reveals distinct similarities and differences across various species. The RGD integrin-binding site in TB4 of all fibrillins is conserved in cephalochordates and vertebrates, while the integrin-binding site within cbEGF18 of fibrillin-3 is a recent evolutionary change. The proline-rich domain in fibrillin-1, glycine-rich domain in fibrillin-2 and proline-/glycine-rich domain in fibrillin-3 are found in all analyzed tetrapod species, whereas it is completely replaced with an EGF-like domain in cnidarians, arthropods, molluscs and urochordates. All collected sequences contain the first 9-cysteine hybrid domain, and the second 8-cysteine hybrid domain with exception of arthropods containing an atypical 10-cysteine hybrid domain 2. Furin cleavage sites within the N- and C-terminal unique domains were found for all analyzed fibrillin sequences, indicating an essential role for processing of the fibrillin pro-proteins. The four cysteines in the unique N-terminus and the two cysteines in the unique C-terminus are also highly conserved.

## Introduction

Fibrillins constitute a family of large extracellular proteins that form the core of highly ordered extended and ubiquitously distributed aggregates, termed microfibrils [1]. Evolutionarily, fibrillins and microfibrils are widely distributed from cnidarians to mammals [2]–[9]. Microfibrils confer structural integrity to individual organ systems and regulate the bioavailability of growth factors of the TGF-β superfamily, including TGF-β and bone morphogenetic proteins [10], [11]. In elastin-expressing vertebrate organisms, microfibrils are believed to provide a scaffold for elastogenesis in blood vessels, lung, skin and other elastic tissues [12]. A number of hereditary connective tissue disorders in humans, including Marfan syndrome, dominant Weill-Marchesani syndrome, geleophysic and acromicric dysplasia, stiff skin syndrome and others are associated with mutations in fibrillin-1, whereas mutations in fibrillin-2 lead to congenital contractural arachnodactyly [13]–[16].

**Figure 1:**
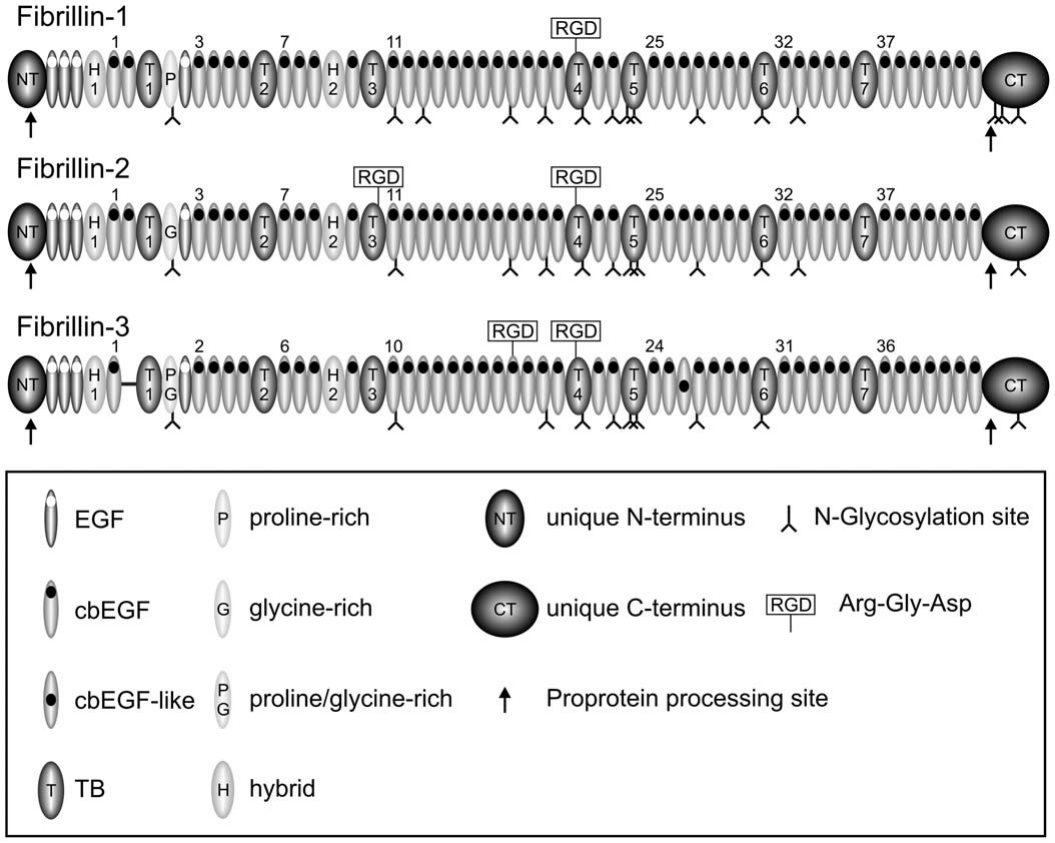
Overview of the domain structure of the human fibrillin family. Numbers above cbEGF domains and within TB and hybrid domains indicate the relative number of the respective domain within the human fibrillin molecule. RGD sites, proprotein processing sites in the unique N- and C-terminal domains (arrows) and predicted N-glycosylation sites (inverted Y symbols) are indicated.

Most of the important structural and functional properties in fibrillins were identified and described with fibrillin nucleotide and protein sequences from mammalian organisms, in particular humans. Thus, the following introductory description of fibrillin properties refers primarily to information available on human fibrillins. The fibrillin family consists of three homologous isoforms, fibrillin-1, -2 and -3. Fibrillins are composed of a typical sequence of individual domains containing between 40–80 amino acid residues (Fig. 1). This characteristic domain signature is 100% conserved between all three fibrillins of mammalian organisms with the exception of an alternatively spliced domain at the N-terminus of fibrillin-3 [17]. Almost all of the fibrillin domains are characterized by a typical number of cysteine residues ranging between 6–9 cysteine residues per domain. Generally, fibrillins are the extracellular proteins with the highest content of cysteine residues (12–13%) [18].

The most prominent domain in fibrillins is the epidermal growth factor-like (EGF) domain which is present 46–47 times in fibrillins. This domain contains six characteristic cysteine residues which form three stabilizing disulfide bonds in a 1–3, 2–4, 5–6 arrangement [19], [20]. The majority of the EGF-like domains contain a consensus sequence ((D/N)X(D/N)(E/Q)X*m*(D/N*)X*n*(Y/F)) for calcium-binding (cb) at their N-terminus where *m* and *n* are variable numbers of amino acid residues, and the asterisk indicates a potential hydroxylation site [21]. Homologous EGF and cbEGF domains are found in numerous extracellular matrix, cell surface and blood proteins throughout all metazoan organisms [19].

Tandem arrays of EGF and cbEGF domains in fibrillins separate two other types of domains, the transforming growth factor (TGF)-β binding protein domains (TB) and the hybrid domains. These two domains are unique to fibrillins and to the latent TGF-β binding proteins (LTBPs) [22]. The TB domains occur seven times in fibrillins and are characterized by eight cysteine residues that form four stabilizing disulfide bonds organized in a 1–3, 2–6, 4–7, 5–8 pattern [23]–[25]. Three of these cysteine residues (Cys3, Cys4, Cys5) are present in a characteristic triple Cys-Cys-Cys motif. Sequence and structural analysis demonstrated that the two hybrid domains present in fibrillins have phylogenetically evolved by fusion of the N-terminus of a TB domain with the C-terminus of a cbEGF domain [7], [26], [27]. Hybrid domains are characterized by a typical Cys-Cys repeat and a 1–3, 2–5, 4–6, 7–8 disulfide-bond pattern [7], [27]. The first hybrid domain in mammalian fibrillins contains an extra cysteine inserted between Cys2 and Cys3. This unpaired cysteine residue were shown to be surface-exposed, suggesting a role in intermolecular disulfide-bond formation [28].

Fibrillins can be structurally distinguished by a characteristic domain without cysteine residues immediately following the first TB domain. This unique domain is rich in proline residues in fibrillin-1, rich in glycine residues in fibrillin-2 and rich in both proline and glycine residues in fibrillin-3 [7]–[9]. For fibrillin-1, the proline-rich region has been suggested as a hinge region facilitating a folding mechanism of the fibrillin molecule in microfibrils [29]. The proline-rich region in fibrillin-1 and possibly the glycine-rich region in fibrillin-2 are involved in the interaction with tropoelastin [30], [31]. The fibrillin N-terminal domain contains four cysteine residues and the C-terminal domain contains 2 cysteine residues. Both terminal domains also contain a tribasic consensus sequence (Arg-Xaa-(Lys/Arg)-Arg) that are recognition signals for proprotein convertases of the Furin/PACE type [32], [33]. Both the N- and the C-terminal domains are proteolytically processed at these sites [28], [34]–[37]. It has been shown for fibrillin-1 that only the processed form becomes incorporated into the extracellular matrix, suggesting that profibrillin-1 processing within its terminal domains plays a regulatory role for its assembly into microfibrils [38]. All three fibrillins in mammalian organisms contain one (fibrillin-1) or two (fibrillin-2 and -3) Arg-Gly-Asp (RGD) sites. Common to all three mammalian fibrillins is a RGD site present in the TB4 domain at the tip of an extensible loop [25]. This site mediates the interactions with αvβ3, α5β1 and αvβ6 integrins [25], [39]–[42]. Human fibrillin-2 and fibrillin-3 contain a second RGD site in TB3 and cbEGF18, respectively. It is not known whether these RGD sites represent functional integrin-binding epitopes. Another differential feature between the three fibrillins is the number and position of predicted N-linked glycosylation sites (see Fig. 1).

Recently, a global evolutionary analysis of TB domain-containing proteins highlighted that the characteristic fibrillin domain organization emerged over 600 million years ago prior to the divergence of cnidarians and bilaterians and before LTBPs emerged [43]. This study further described the unpaired cysteine residue in the first hybrid domain, as well as the CXXC motif in the C-terminus as absolutely conserved between species, while the RGD site in TB4 is not conserved outside vertebrates.

In the present study we have retrieved and extensively curated 78 fibrillin gene and protein sequences from 39 organisms ranging from cnidarians to mammals. We have developed evolutionary trees suggesting that a single ancestral fibrillin gene still present in invertebrates and jawless fish (agnathans) underwent duplication. One of the resulting genes evolved to become fibrillin-1, while the other underwent evolutionary changes, including a second duplication, to produce fibrillin-2 and fibrillin-3. We have further analyzed the evolution of critical functional motifs and domains in fibrillins.

## Methods

### Sequence Collection

Fibrillin nucleotide and protein sequences for 39 organisms were sequentially retrieved from publically available databases including the DOE Joint Genome Institute (http://www.jgi.doe.gov/), Ensembl (http://uswest.ensembl.org/), the National Center for Biotechnology Information nucleotide database (http://www.ncbi.nlm.nih.gov/nuccore/), the UCSC Genome Bioinformatics (http://genome.ucsc.edu) and the EMBL European Bioinformatics Institute (http://www.ebi.ac.uk). A complete list of retrieved sequences and the corresponding databases is shown in Table S1. Keyword searches with the terms “fibrillin,” “fbn,” “fbn-like,” and “similar to fibrillin” were used to find annotated fibrillin sequences. In order to identify non-annotated fibrillin sequences, BLASTP, TBLASTN and BLASTN searches were conducted using sequences of characteristic fibrillin domains as queries, including the TB and the hybrid domain [44]. FASTA comparisons with known fibrillin sequences were used on incomplete or uncertain non-annotated sequences in order to distinguish between fibrillins and LTBPs [45], [46]. Retrieved sequences were stored in a GCG software package version 11.1 database (Accelrys). Following a complete sequence alignment with ClustalX 2.0 [47], minor sequence adjustments were made to remove missense insertions, correct missense regions and fill in gap regions with the appropriate sequence. Missense insertions were only removed if they were located between intron/exon boundaries. Adjustments were made based on evidence from the genomic scaffolds or contigs used to derive the original sequences.

### Manual Sequence Reconstruction

Many of the protein database entries are predicted by automated computation from genomic nucleotide sequences. As a consequence, certain sequences were either incomplete or appeared to contain errors such as insertions and deletions due to misinterpretations of intron/exon boundaries and open reading frames. To compare these critical regions with other sequences, corresponding raw genomic chromosomes, scaffolds or contigs were downloaded from databases and translated into all six reading frames. Upon determining the appropriate direction, the three corresponding reading frames were simultaneously viewed and a known fibrillin sequence was used as a template to highlight and extract individual domains from the raw sequence. With this method, we successfully reconstructed six complete fibrillin sequences, as well as filled in partial or missing regions for various other sequences. For some sequences, precise intron/exon boundaries were difficult to determine and as a consequence, the sequences around certain intron/exon boundaries are uncertain. However, by analyzing these reconstructed sequences with ClustalX, we confirmed the validity of the sequences based on homology. A complete list of manually reconstructed and modified sequences is provided as part of Table S1.

### Phylogenetic Analysis

Given that signal peptides are often species-specific [48], they were identified using the SignalP 3.0 server and then removed from the sequences (http://www.cbs.dtu.dk/services/SignalP) [49]. In cases with low signal peptide cleavage site probability, sequence homology was included as a further criterion. A total of 78 protein sequences from 39 organisms were used in the analysis. A multiple sequence file was generated using GCG's ClustalW+ program. A complete alignment was then conducted on the resulting file using ClustalX 2.0 with default settings. Gap-only columns were subsequently removed. Using the ProtTest-HPC 3.0 objective model testing program with Akaike Information Criterion (AIC), the Jones-Taylor-Thornton (JTT) model with invariant sites and a gamma distribution was determined to be the most appropriate for the given alignment (ΔAIC = 0.00, AICw = 1.00) [50], [51]. Maximum likelihood phylogenetic reconstructions were generated with this substitution model using Molecular Evolutionary Genetics Analysis (MEGA) 5.05 software [52]. The Nearest-Neighbor-Interchange (NNI) search method was employed. Reliability of the trees was evaluated using 1,000 bootstrap repetitions. Similar results (not shown) were obtained using the JTT substitution model with the Randomized Axelerated Maximum Likelihood (RAxML) program (version 7.2.8) on the CIPRES Portal (http://www.phylo.org/sub_sections/portal) [53], [54]. Distance-based phylogenetic trees were constructed using a series of four sequentially executed programs from the PHYLIP version 3.69 software package [55]. First, “Seqboot” was used to generate 1,000 data sets from the alignment file in the PHYLIP format. Next, “Protdist” was used to generate a distance matrix using the JTT model. Using the program “Neighbor,” we then constructed phylogenetic trees from each data set using the Neighbor-Joining method [56]. Finally, the program “Consense” was used to generate the consensus tree.

Phylogenetic trees for fibrillin proteins were initially generated in the following three principal ways. Separate distance-based phylogenetic reconstructions were computed for each of the seven TB domains; sequences with gaps or apparent missense regions in these corresponding domains were not included in the analysis. This allowed us to determine the evolutionary history of each individual TB domain and to include the largest number (59–69) of fibrillin sequences in the analysis. An additional distance-based phylogenetic tree using as input the seven concatenated TB domains was also generated in order to calculate an “average” tree. However, since not all fibrillin sequences are completely available, the total number of fibrillin sequences in this analysis was reduced to 48. A final distance-based phylogenetic tree was generated for the entire sequence using 37 fibrillins; partial and highly-gapped sequences were not included in the analysis. The second TB domain from human LTBP-2 was used as the outgroup for the seven individual fibrillin TB domain analyses. With regards to the tree generated from the seven concatenated TB domains, the same outgroup was used, however it was duplicated seven times to generate a comparable 7-TB-domain structure. Alternatively, the phylogenetic analyses of the seven concatenated fibrillin TB domains were performed with an outgroup consisting of the concatenated individual three TB domains found in human LTBP-2 (not a repeated single TB domain). This method produced very similar results (not shown). The full human LTBP-2 sequence was used as the outgroup for the tree generated from the complete fibrillin sequences. Phylogenetic reconstructions were repeated using the maximum likelihood method for both the concatenated TB domains sequence and the complete fibrillin sequence. Non-fibrillin outgroup sequences were omitted. Final consensus trees were re-rooted around the base of the single fibrillin clade. Similar results were obtained from an additional maximum likelihood phylogenetic reconstruction of the concatenated TB domains using the corresponding 7-TB-domain human LTBP-2 sequence as an outgroup.

### Analyses of Short Sequence Motifs

The FindPatterns program from the GCG software package was used to search for matching and homologous short sequence motifs, including integrin-binding sites and furin-cleavage sites. The pattern RGD was used to identify RGD-dependent integrin-binding sites. Furin-cleavage sites were located using the pattern RX(K,R)R, interpreted as arginine followed by any amino acid, followed by either lysine or arginine, followed by arginine.

## Results and Discussion

We have retrieved 78 fibrillin sequences from a total of 39 organisms representing several taxonomic groups, including chordates, arthropods, annelids, echinoderms, molluscs and cnidarians from publically available databases (Table S1). After careful inspection and correction of sequence errors as explained in detail in Material and Methods, we performed phylogenetic analyses and studied the evolution of known key functional sequences. Only high quality sequences were included in the individual analyses, whereas regions of low quality sequences were not included. Results and discussions that include domain numbers use the numbering system of human fibrillin-1 as a reference (Fig. 1).

### Phylogenetic Analysis

All fibrillins from all species are characterized by a typical fibrillin domain signature containing seven isolated TB and one or two hybrid domains interspersed by characteristic numbers of cbEGF domains, as originally identified in human fibrillins (Fig. 1). As expected, all mammals, except rodents, armadillo and platypus, have three functional fibrillin genes. It was previously described that the rodent fibrillin-3 gene is inactivated, potentially due to chromosome fission events [17]. A fibrillin-3 database entry was found for guinea pig, however several non-homologous insertions were found throughout the sequence. With the present data, we are unable to determine if these insertions are in fact present *in vivo*, thus yielding a non-functional transcript consistent with other rodents, or if these regions merely reflect sequencing errors. The lack of fibrillin-3 in armadillo and platypus may be due to either incomplete sequencing of these genomes or species-specific gene losses. Three fibrillin genes were also identified in chicken, zebra finch, lizard, frog and zebrafish (see Text S1 for more information on zebrafish fibrillins). Other ray-finned fish (actinopterygian) species, including the Japanese pufferfish, the spotted green pufferfish and the medaka fish, contain two fibrillin genes. A single fibrillin was found in species representative of several phyla and subphyla, including cnidarians, molluscs, annelids, arthropods, echinoderms, urochordates, cephalochordates and vertebrates. The single fibrillin found in vertebrates is restricted to agnathans. An overview of the fibrillin genes and proteins identified and analyzed is presented in Table S1.

**Figure 2:**
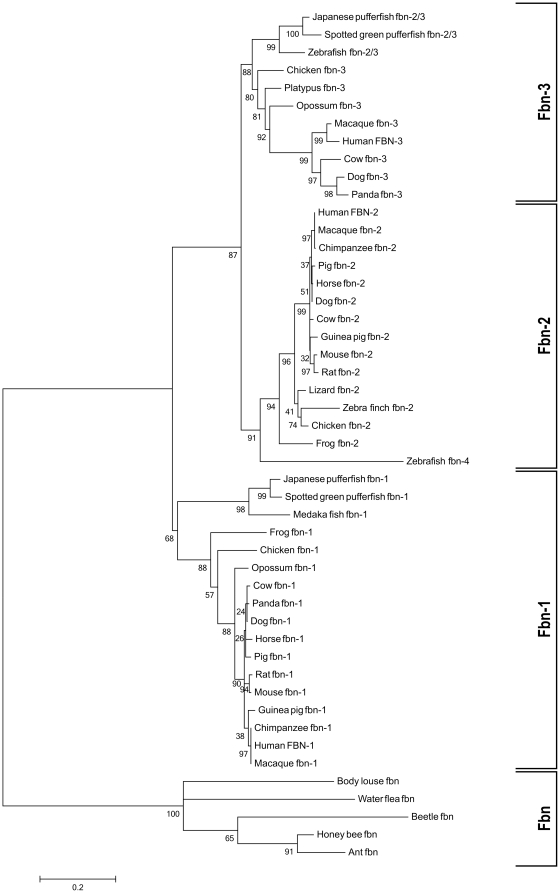
Maximum likelihood phylogenetic tree generated from seven concatenated TB domains of each fibrillin. The fibrillin protein family is grouped into four separate clades (from bottom to top): single fibrillin (Fbn), fibrillin-1 (Fbn-1), fibrillin-2 (Fbn-2), and fibrillin-3 (Fbn-3). Bootstrap values shown on each node represent the percentage of trees (out of 1,000) yielding the same two-set partition of sequences. The Jones-Taylor-Thornton model with invariant sites and a gamma distribution with four discrete categories was used. The tree was re-rooted around the base of the single fibrillin clade. The scale bar indicates the estimated average number of expected substitutions per site. Analyses of full length fibrillins or of full length fibrillins lacking the unique region resulted in similar phylogenetic trees.

Phylogenetic trees were generated with the entire fibrillin sequences, with the seven concatenated TB domains from each fibrillin and with the individual TB domains. Generally, all generated phylogenetic trees support the existence of an ancestral fibrillin gene, consistently placed outside of the fibrillin-1, -2, and -3 clades, and corresponding with species containing a single fibrillin gene (Fig. 2). While a single fibrillin sequence was found in several insects, including the honey bee, ant, beetle, louse and aphid, none was found in the fruit fly (*Drosophila melanogaster*). Furthermore, contrary to existing database entries annotated as fibrillin homologs in the nematode *Caenorhabditis elegans* (NCBI: NM_066269.2; Ensembl: Y64G10A.7), the absence of the typical fibrillin domain signature, including TB and hybrid domains, does not support the existence of fibrillin homologs in *C. elegans*, nor in several other nematode species. The same rationale applies to database entries annotated as putative fibrillin sequences in *Schistosoma mansoni*, a trematode (EnsemblMetazoa: Smp_167190, Smp_067800). These findings are in agreement with the previous report by Robertson *et al.* and suggest that the fibrillin gene was either lost or severely disrupted during the evolution of these organisms [43].

All distance-based and maximum likelihood phylogenetic reconstructions support the concept of two gene duplications as a means of generating the three present-day fibrillin sequences from a single ancestral fibrillin (Fig. 2, Table 1). The ray-finned fish species surveyed in this analysis, including the zebrafish, Japanese pufferfish, medaka fish, and the spotted green pufferfish, are the oldest taxonomic groups found to contain more than one fibrillin gene. The presence of only a single fibrillin gene in invertebrates, including the sea squirt (*Ciona intestinalis*), amphioxus (*Branchiostoma floridae*), and the agnathan sea lamprey (*Petromyzon marinus*) strongly suggests that the first fibrillin gene duplication occurred prior to the divergence of ray-finned fish (Fig. 2, Table S1). This gene duplication resulted in a fibrillin-1 gene that significantly evolved further to become the gene expressing present-day fibrillin-1 (Fig. 2). The second gene, termed fibrillin-2/3, underwent further evolutionary changes, including a second duplication, to produce the genes for present-day fibrillin-2 and fibrillin-3 (Fig. 2). This second duplication appears to coincide with the divergence of actinopterygians and tetrapods, and is supported by the presence of three fibrillin genes in the amphibians, as well as in all analyzed reptile, bird and mammalian species. In considering the distance-based phylogenetic trees, the bootstrap values for the initial gene duplication leading to fibrillin-1 and fibrillin-2/3 (100% for concatenated TB domains and 99.9% for the complete protein) and for the second gene duplication to form separate fibrillin-2 and fibrillin-3 genes (100% for concatenated TB domains and 96.7% for the complete protein) strongly support this conclusion (Table 1). Bootstrap values obtained from the analyses with the individual TB domains are typically more variable, as expected for shorter sequences. For the first gene duplication, high bootstrap values are obtained for TB1, TB2, TB3 and TB6 (76.5–94.3%), while TB4, TB5 and TB7 showed lower bootstrap values (45.4–53.2%). Generally, these values provide additional support for the initial gene duplication. Bootstrap values from individual TB domains for the second split are generally relatively low (21–47.5%) for all TB domains except for TB2 (66.5%). However, this gene duplication hypothesis is strongly supported by the maximum likelihood phylogenetic reconstructions for the concatenated TB domains (100% for the first duplication, 87% for the second duplication) and the complete fibrillin sequences (100% for the first duplication, 100% for the second duplication). In summary, while the distance-based phylogenetic reconstructions from each individual TB domain do not always provide high bootstrap values in support of the duplication events, the analysis of the trees generated from the concatenated TB domains and the complete fibrillin sequences using both distance-based and maximum likelihood methods provides sufficient evidence to support the series of two gene duplications to yield three present-day fibrillins from one ancestral fibrillin.

**Table 1:** Comparison of bootstrap values.

	TB1	TB2	TB3	TB4	TB5	TB6	TB7	Concatenated TB domains (DB/ML)	Complete Protein (DB/ML)
**FBN1∶FBN2/3 split**	94.3	98.0	80.5	46.4	45.4	76.5	53.2	100/100	99.9/100
**FBN2∶FBN3 split**	47.5	66.5	31.4	21.0	26.3	44.4	29.8	100/87	96.7/100
**Number of sequences**	59	65	62	67	60	65	69	48	37

Bootstrap values, that is, the percentage of trees yielding the same two-set partition of sequences for a given node, are indicated in percentages (two top rows). Bootstrap values were generated from 1,000 data sets. Distance-based phylogenetic trees were generated with individual TB domains (TB1–TB7). Distance based (DB) and maximum likelihood (ML) based methods were generated with all TB domains of a fibrillin protein concatenated together and with the entire fibrillin sequences as indicated. The numbers of fibrillin sequences included in the respective analyses are indicated in the bottom row.

### Analysis of Functional Regions

#### RGD Integrin-Binding Sites

**Figure 3:**
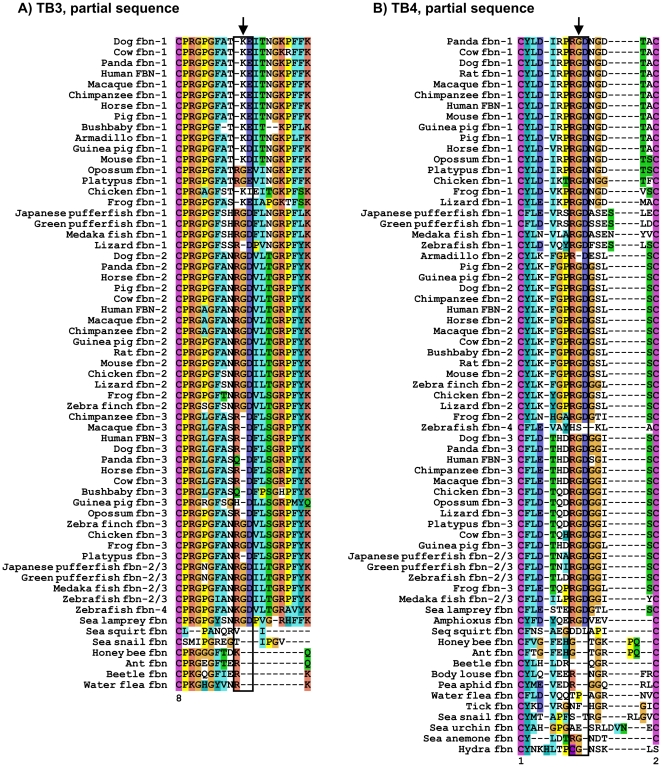
ClustalX alignment of RGD cell interaction sites in A) TB3 and B) TB4. Only the relevant loop containing the RGD sequence is shown. RGD sequences are highlighted by a box and an arrow. The relative numbers of the cysteine residues within the respective TB domain is indicated on the bottom. Note for TB3 the presence of a RGD integrin-binding site in sea lamprey, ray-finned fish fibrillin-1 and fibrillin-2/3, all fibrillin-2 species and several tetrapod fibrillin-3 sequences. For TB4, the RGD integrin-binding site is present in all vertebrate fibrillins, with the exception of armadillo fibrillin-2. Invertebrate fibrillin sequences do not contain an integrin-binding site in TB4.

A characteristic feature that distinguishes amongst the fibrillin isoforms in human and other mammals is the differential presence and location of cell surface integrin-binding sites, characterized by the amino acid sequence RGD (Fig. 1). All three forms of human fibrillin contain a RGD site within TB4, while fibrillin-2 and fibrillin-3 have an additional RGD site located in TB3 and cbEGF18, respectively [57]. Analysis of the evolutionary conservation of these sites confirmed that all vertebrate fibrillin-1, -2, and -3 sequences contain a RGD site within TB4, located between Cys1 and Cys2 of this TB domain (Fig. 3B) [43]. The single fibrillin found in both sea lamprey (jawless fish) and amphioxus (cephalochordate) also has a RGD site in this domain. The presence of a RGD site in TB4 of a cephalochordate contradicts recent findings and emphasize that this site is not strictly limited to vertebrates [43]. Furthermore, sea lamprey contains a RGD site in TB3, while in amphioxus, the binding site is incomplete, although sequence quality in this region is lower than in other regions (Fig. 3A). The fibrillin sequence found in the urochordate *Ciona intestinalis* does not have a RGD site in either of these domains. The single fibrillin found in other invertebrates, including cnidarians, arthropods, molluscs and echinoderms, does not have a RGD site in TB3 or TB4. Thus, the introduction of RGD integrin-binding sites in both TB3 and TB4 occurred during the evolution of chordates, prior to the emergence of sea lamprey and possibly even before that of amphioxus.

With respect to fibrillin-3, only human and chimpanzee fibrillin-3 have RGD sites in cbEGF18, while KGD is found in this domain in the Rhesus macaque, a primate, and NAD, NAE, NAN, or RAD are found in other fibrillin-3 sequences. This finding strongly suggests that the presence of an integrin-binding site in fibrillin-3 within cbEGF18 is a very recent evolutionary change. It is currently not known whether the RGD site in cbEGF18 of human and chimpanzee fibrillin-3 is functional with respect to cell binding.

The integrin-binding site analysis further revealed additional characteristics of the fibrillin family as it evolved over time. Integrin-binding sites were found in TB3 of fibrillin-1 and fibrillin-2/3 sequences, at the homologous fibrillin-2 RGD site, for all surveyed ray-finned fish species (Fig. 3A). The fibrillin-3 sequences for the chicken and the frog also contain a RGD site in TB3. The platypus (monotreme) and opossum (marsupial) fibrillin-1 sequences contain the residues RGE, suggesting that the loss of the integrin-binding site within TB3 of fibrillin-1 occurred after the divergence of ray-finned fish but prior to the evolution of mammals. Within monotreme and marsupial fibrillin-3 sequences, the glycine residue is absent from the integrin-binding site, a feature that is maintained in more recently diverged groups such as placental mammals (eutherians). Therefore, we conclude that prior to the initial gene duplication, the ancestral fibrillin protein contained integrin-binding sites in TB3 and TB4, as supported by the presence of these sites in ray-finned fish fibrillin-1 and fibrillin-2/3. The fibrillin-1 RGD site in TB3 was lost prior to the evolution of amphibians, while the RGD site in TB3 of fibrillin-2/3 remained even after the second gene duplication, where it is conserved in all fibrillin-2 sequences, as well as in fibrillin-3 sequences of amphibians and birds. As the fibrillin-3 sequence continued to change, the evolution of mammals brought with it the loss of the RGD site in TB3, and only the most recent fibrillin-3 sequences, including that of humans and chimpanzees, experienced the addition of an integrin-binding site within cbEGF18. Therefore, we propose that the integrin-binding sites of fibrillin-2, found within TB3 and TB4, are the most conserved and representative of the RGD sites in the single fibrillin protein that exists in cephalochordates and agnathans. Thus, the presence of integrin-binding sites is highly conserved in chordates, although gene duplication allowed for the loss of the binding sites in TB3 in fibrillin-1 and -3. In addition to these RGD sites, which provide insight into the evolutionary relationship of fibrillins, several other RGD sites were noted in various fibrillin domains of organisms containing only one fibrillin gene. For completeness, these sites are summarized in Table S2.

#### Analysis of the Unique Region

**Figure 4:**
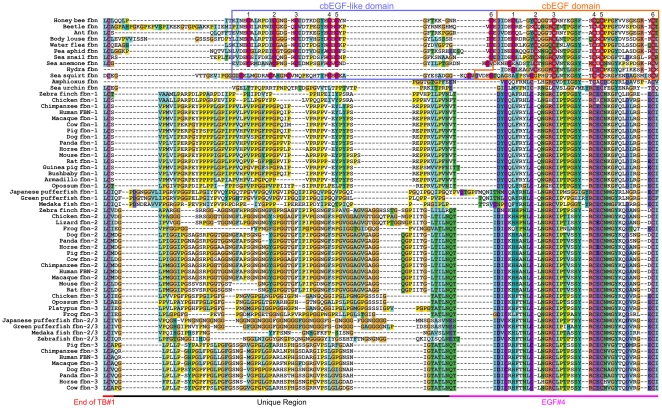
Excerpt from the ClustalX alignment of full length fibrillin focusing on the unique regions and the subsequent EGF4 domain. The end of TB1, the unique region, and the EGF4 domain found characteristically in mammalian fibrillins are indicated on the bottom. For invertebrate organisms, the cbEGF-like (purple box), and the new cbEGF domain (orange box) are highlighted, and cysteine residues are circled in red in these domains. The relative numbers of the cysteine residues within the respective EGF domain is indicated on top. Note that the unique region does not exist in invertebrate fibrillin proteins, and is instead replaced by a cbEGF-like domain. The non-calcium binding EGF4 domain that typically follows the unique region is replaced in invertebrate fibrillins by a cbEGF domain.

Fibrillin isoforms in humans and other mammals can also be distinguished by a characteristic unique domain located towards the N-terminus of the protein immediately following the TB1 domain. In humans and other mammals, fibrillin-1 is characterized by a proline-rich region, fibrillin-2 by a glycine-rich region and fibrillin-3 by a mixed proline-glycine-rich region. Here, we collectively use the term “unique region” to generally address this domain in all fibrillin isoforms. Figure 4 shows an excerpt from the ClustalX alignment of the complete fibrillin sequences, highlighting the unique region. While all domains upstream and downstream of this region are completely aligned and characterized by high homology between the species, the overall homology within the unique region between all species is much lower. In the following, we discuss several aspects of this alignment.

As expected, the alignment shows clustering of the three vertebrate fibrillin isoforms into proline-rich (fibrillin-1), glycine-rich (fibrillin-2) and proline-glycine-rich (fibrillin-3) domains. Regarding fibrillin-1, the overall structure of the proline-rich region is relatively well-conserved within tetrapod species, while in all ray-finned fish species, this region begins with a 10-residue insert and ends with an additional insert of variable length. In fibrillin-2, the chicken, zebra finch and the lizard all contain a glycine-rich region that varies slightly in comparison to mammalian fibrillin-2 sequences. The proline-glycine-rich region in fibrillin-3 shows a differential structure, whereby the surveyed tetrapod species, including the chicken, opossum, platypus and frog, all contain a unique region that more closely resembles that of fibrillin-2. In mammalian species that contain a functional fibrillin-3 gene, including humans, the structure of this unique region is significantly altered and includes numerous amino acid sequence changes, in addition to the deletion of a 13-residue segment near the C-terminal portion of this domain. Thus, by demonstrating that the tetrapod fibrillin-3 sequences found in amphibians, birds, marsupials and montremes contain unique regions that more closely resemble that of fibrillin-2, with significant evolutionary changes only present in eutherians, we confirm the hypothesis that fibrillin-2 and fibrillin-3 are paralogues.

In an effort to better characterize the difference in residues amongst the three fibrillin isoforms, the unique regions of all collected high quality sequences were examined with respect to the on average most commonly occurring amino acids (Table 2). As expected, the analysis for fibrillin-1 revealed that proline (40%) is the most common amino acid, followed by valine (14%). For fibrillin-2, glycine (38%) is the most common amino acid and proline (12%) is the second-most common. In the fibrillin-2/3 sequences for the ray-finned fish species, glycine (46%) is the most common residue and asparagine (16%) is the second-most common residue. Within the unique region of the pre-eutherian fibrillin-3 sequences, glycine (31%) and proline (20%) are the most common and second-most amino acids, respectively. In the eutherian fibrillin-3 unique region, glycine (24%) is the most common residue, while the second-most common amino acid varies between either proline (18%) or identical amounts of proline and leucine (16%). These results indicate that a glycine-rich region, similar to that found in fibrillin-2, is already present in fibrillin-2/3, further supporting the hypothesis that fibrillin-2 and -3 are paralogues. Fibrillin-3 sequences in tetrapod species that diverged prior to placental mammals are characterized by a lower glycine∶proline ratio in comparison to fibrillin-2. This ratio decreases even further in eutherian fibrillin-3. While chimpanzee and human fibrillin-3 sequences do maintain this low glycine∶proline ratio, they are also characterized by an equal number of leucines and prolines as the second-most common amino acid. Based on these relative amounts of the most common amino acid residues, we speculate that the unique regions in all three present-day fibrillins serve different functions.

**Table 2:** Amino acid composition of the unique region.

Sequence	Most common amino acid (number of sequences/percentage of unique region)	Second-most common amino acid (number of sequences/percentage of unique region)
**Fibrillin-1**	Proline (18/40%)	Valine (14/14%)
**Fibrillin-2/3**	Glycine (3/46%)	Asparagine (3/16%)
**Fibrillin-2**	Glycine (15/38%)	Proline (14/12%)
**Pre-eutherian fibrillin-3**	Glycine (4/31%)	Proline (4/20%)
**Fibrillin-3**	Glycine (7/24%)	Proline (4/18%)Leucine & Proline (3/16%)

The most common and second-most common amino acids are shown for each of the 3 modern fibrillins, as well as for ray-finned fish fibrillin-2/3 and pre-eutherian fibrillin-3. The use of the ampersand symbol (&) indicates that both amino acids occur at the same frequency.

Surprisingly, the analysis of the region following TB1 revealed the absence of a characteristic unique region from all organisms containing a single fibrillin sequence. It should be noted, however, that we could not determine with confidence the absence of the unique region in hydra, sea urchin, lamprey and amphioxus due to sequence uncertainties in this region. The sequence of amino acids immediately downstream of TB1 does not contain any prevalent amino acid. Domain analysis reveals that this region consists of a linker followed by an EGF domain. Figure 4 shows the ClustalX alignment for this region in ancestral fibrillins in the context of the vertebrate fibrillin unique regions. This EGF domain contains six characteristically spaced cysteines, as well as all important residues for calcium-binding, except for the first residue of this domain, which differs from the typical calcium-binding consensus sequence in that it is not the required aspartic acid or asparagine residue [20], [21]. Since this critical amino acid residue for calcium binding is not present at this position, we annotate this domain as a cbEGF-like domain. Furthermore, given the absence of this residue from each of these sequences, we argue against the possibility of a sequencing error or codon split across an intron/exon boundary. Of further interest, immediately downstream of this cbEGF-like domain, the single fibrillin sequences have a *bona fide* cbEGF domain replacing the (non-calcium-binding) EGF4 domain that typically follows the unique regions in mammalian fibrillins. Thus, the single fibrillin sequences found in protostomes and urochordates contain a cbEGF-like domain, followed by a true cbEGF domain in place of the unique region and the fourth EGF domain, whereas in echinoderms and cephalochordates, this last domain begins to resemble the EGF4 domain found in vertebrate fibrillins.

It was speculated that the proline-rich region in human fibrillin-1 fulfills a flexible hinge-region that may be critical for folding of fibrillin-1 into highly ordered microfibrils [29]. Although no fine structure of microfibrils from invertebrate organisms is available, it is clear that beaded microfibrils, very similar to those found in vertebrates, are also found in invertebrate organisms including, sea cucumber (*Cucumaria frondosa*), lobster (*Homarus americanus*), and jellyfish (*Podocoryne carnea*) [2]–[4]. The pair of cbEGF-like domain followed by a cbEGF domain in invertebrate organisms is expected to adopt a relatively stiff rod-like structure, lacking a similar hypothetical flexibility of the unique regions [20], [58], [59]. However, the presence of a non-calcium binding cbEGF-like domain following TB1 domain suggests that this linker region lacks a typical TB-EGF interdomain interaction and thus still may fulfill a critical flexible role for the folding of fibrillin-1 into microfibrils.

The proline-rich region in human fibrillin-1 may be involved in the interaction with tropoelastin [31]. In addition, a small N-terminal fragment containing the glycine-rich region of human fibrillin-2 was demonstrated to interact with tropoelastin [30]. It is not known whether the unique region in fibrillin-3 plays a similar role. Elastin emerged in evolution with the appearance of a closed circulatory system and is exclusively found in vertebrates except for agnathans, including lamprey [43], [60]. The evolution of the unique region in vertebrate fibrillins coincides with or is followed by the appearance of elastin and the first gene duplication of the single fibrillin into fibrillin-1 and fibrillin-2/3. A speculative interpretation of this data is that the unique regions were evolutionarily invented to support the interaction of fibrillins with tropoelastin, and that this interaction may require more than one fibrillin. If this is true, then the fibrillin gene duplication may have been a driving force in the development of closed circulatory systems. Additional support for this hypothesis comes from the genetic deletion of both, fibrillin-1 and -2 in mouse, which had much more severe effects on the structural integrity of elastic fibers in the aortic wall compared to animals with only a single fibrillin deleted [61], [62].

#### Hybrid Domains

The first hybrid domain in human fibrillin-1 and -2 has been demonstrated to contain a free cysteine located on the surface of the domain and was thus suggested to contribute to potential intermolecular disulfide bonds [28]. When primary sequences of hybrid domains in fibrillins where first analyzed, it was noted that they combine sequence homology at their N-termini with TB domains and at their C-termini with EGF domains [7], [26]. More recently, a high resolution structure of the human hybrid 2 domain demonstrated that these similarities in the primary sequence are fully reflected in the three-dimensional structure of this domain, proving that the hybrid domain is indeed an evolutionary “hybrid” between TB and cbEGF domains [27].

All high quality fibrillin sequences contain the first 9-cysteine hybrid domain, confirming previous findings [43]. The only exception was the absence of the extra cysteine from dog fibrillin-3. Given that all other collected sequences contain this additional cysteine, we suspect that this is likely due to a sequencing error. The additional cysteine is always positioned after cysteine 2 in the hybrid 1 domain. The amino acids upstream and downstream of this extra cysteine are also highly conserved amongst all fibrillin-1 and -2 sequences, as well as in tetrapod fibrillin-3 sequences that diverged prior to placental mammals. Single fibrillin and vertebrate fibrillin-3 sequences, while still well-conserved in this region, do contain several non-conserved residue changes. The evolutionary conservation of the nine cysteine pattern suggests that the exposed cysteine plays a critical function in fibrillins. Provided the surface location determined in human fibrillin-1 and -2, this cysteine may indeed participate in intermolecular disulfide bonds, perhaps to stabilize staggered bundles of microfibrils [63].

**Figure 5:**
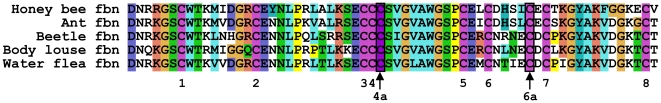
ClustalX alignment of the second hybrid domain in arthropods. Relative numbering of the cysteine residues in the hybrid 2 domain as identified in human fibrillins is indicated on the bottom. In arthropods, this domain has two additional cysteine residues (black boxes indicated by arrows) annotated here as cysteine 4a and 6a.

The hybrid domain 2 of all analyzed organisms including cnidarians, but excluding arthropods, contains 8 cysteines with the typical tandem Cys-Cys in the relative 4–5 position. While the relevant domain in arthropods has considerable homology with typical hybrid domains, it contains 10 cysteines instead of eight as observed previously by Robertson et al. (Fig. 5) [43]. Interestingly, in this hybrid domain 2, an additional cysteine (annotated as Cys4a) extends the hybrid domain typical Cys-Cys motif into a typical triple Cys-Cys-Cys motif found in TB domains. A second additional cysteine (annotated as Cys6a) just prior to the penultimate cysteine renders the C-terminus of this domain highly homologous to the C-terminus of an EGF domain with the consensus sequence -C-X-C-X-X-G-X_8_-C-X (Fig. 5) [7]. The functional implication of these two additional cysteines is unknown. In summary, the data indicates that the hybrid domain 2 in the ancestral fibrillin was present as an 8-cysteine hybrid domain. The 10-cysteine hybrid-like domain found in arthropods, specifically within hexapods and crustaceans, appears to have evolved later in this group.

#### Proprotein Processing Sites

Cleavage of the tribasic proprotein processing sites located in the unique N- and C-terminal domains of fibrillins have been described, based on experiments with human cells, as a regulatory mechanism of fibrillin-1 assembly into microfibrils [38]. Furin-type proteases cleave fibrillins at the consensus motif Arg-Xaa-Lys/Arg-Arg during secretion or immediately thereafter [32], [33], [64]. In the present analyses, furin-cleavage sites were found within both the N- and C-terminal unique domains for almost all high-quality fibrillin sequences. For these fibrillin sequences, the furin-cleavage sites completely matched the consensus sequence. Only three exceptions to the consensus sequence were found in the following sequences: chimpanzee fibrillin-3 N-terminus (RVWR), cow fibrillin-3 N-terminus (SVRR), and guinea pig fibrillin-3 C-terminus (RPPR) (see comment above about guinea pig). While the regions surrounding the cleavage sites for these sequences appear to correspond with other organisms, these exceptions may be due to sequencing errors, as in all three cases, a single nucleotide change can alter the respective codon to restore a fully compatible furin-cleavage consensus site. In summary, the strong conservation of both the N- and the C-terminally located furin consensus site indicate that these sites are critical to the production of functional microfibrils throughout all species analyzed in this study. The presence of beaded microfibrils in various tissues of some invertebrate organisms including sea cucumber, lobster and jellyfish further support this conclusion [2]–[4].

#### Other Sequence Analyses

**Figure 6:**
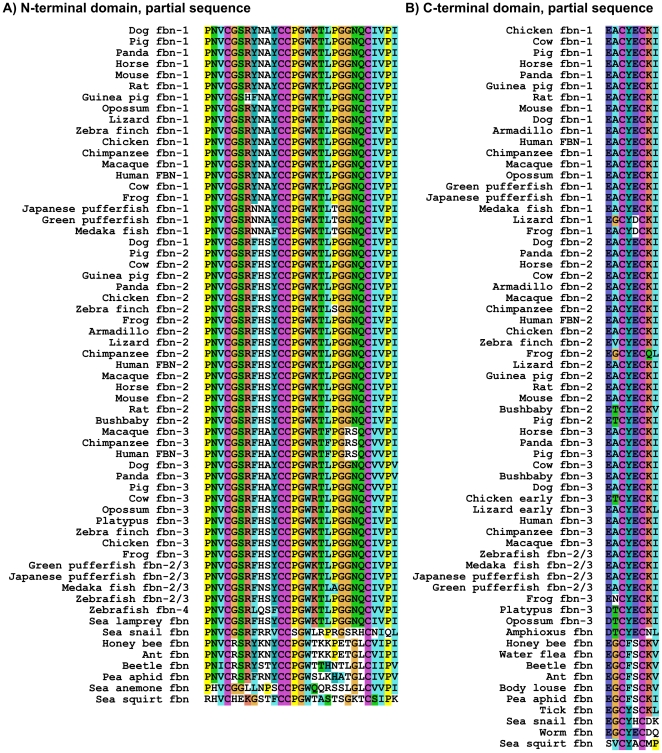
ClustalX alignment of the regions containing cysteines in the unique A) N- and B) C-terminal domains. Note the high conservation of the six cysteine residues and the surrounding sequences in both domains.

The unique N- and C-terminal fibrillin domains of human and other mammalian organisms contain four and two cysteines, respectively. The status of these cysteines with regards to disulfide-bond patterns is not known. Analyses of evolutionary conservation of those cysteines showed that all six cysteines are completely conserved in all high quality fibrillin sequences, suggesting that they have crucial functions in fibrillins (Fig. 6). Furthermore, amino acids in close proximity to these cysteines are also highly conserved.

It has been shown that two cysteine residues in the penultimate TB domain of human LTBP-1, 3 and 4 form covalent bonds with the latency associated protein that keeps TGF-β in its inactive state [24], [65]. A two-amino acid insertion between cysteines 6 and 7 of this particular TB domain is absolutely critical for this interaction, a feature that is absent in other TB domains of LTBPs or human fibrillins [24], [65]. Robertson *et al.* found that this insertion is conserved in the homologous TB domain of all LTBP-like proteins analyzed in their study [43]. Inspection of all relevant and available fibrillin TB domains in the present study revealed the absence of a similar two amino acid insertion between cysteine 6 and 7, confirming that this insertion only occurred in LTBPs.

### Conclusions

**Figure 7:**
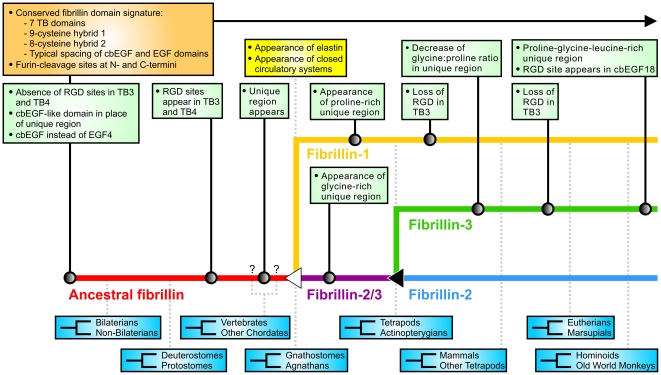
Hypothetical evolution of human fibrillins. An ancestral fibrillin (red) existed prior to the split of bilaterians from non-bilaterians. A gene duplication event (white triangle), presumably at the divergence of gnathostomes (jawed vertebrates) from agnathans (jawless fish), led to the genes for fibrillin-1 (orange) and the ancestral fibrillin-2 and -3 gene, coined as fibrillin-2/3 (purple), and further coincides with the appearance of elastin and the evolution of closed circulatory systems. A second duplication event (black triangle), coinciding with the evolution of tetrapods, led to the genes for fibrillin-2 (blue) and fibrillin-3 (green). The pre-eutherian fibrillin-3 gene underwent significant evolutionary changes to become present-day fibrillin-3. Taxonomic classifications and evolutionary divergence events are found in blue boxes and indicated by fork symbols, with dotted light gray connectors to the individual fibrillins. The upper classification shares a common ancestor with humans. Question marks indicate uncertainties as to whether the unique region appeared before or after the divergence of vertebrates from other chordates. Spacing between taxonomic classifications is not proportional to their evolutionary timeline. Identified conserved characteristic features and evolutionary events found in all analyzed fibrillins are highlighted in an orange-colored box with a black arrow. Evolutionary characteristics and events specific to individual fibrillins are shown in green boxes with solid connectors and circles. Important related evolutionary events are shown in a yellow box.

Using results from the conducted analysis, we studied the evolution of fibrillins throughout key points in evolution. The earliest form of an ancestral fibrillin sequence, still present in cnidarians, molluscs, annelids, arthropods, echinoderms, urochordates, cephalochordates and the vertebrate sea lamprey, already represents the majority of the characteristic domain pattern seen in human fibrillins. Notable differences include the lack of a unique region, replaced by a cbEGF-like domain followed by another cbEGF domain instead of EGF4, the absence of integrin-binding sites until the emergence of cephalochordates and agnathans, as well as the presence of an ancestral hybrid-like domain in place of the second hybrid domain in arthropods. At the time of the first gene duplication, which coincides with the divergence of jawed vertebrates and agnathans, the single fibrillin sequence had already acquired a unique region, the second mature hybrid domain and RGD sites in TB3 and TB4. The coincidence of the first gene duplication and the appearance of the unique regions with the evolutionary appearance of elastin highlight potentially important functions of the unique regions and the availability of more than one fibrillin for the development of elastic fibers and a closed circulatory system. Following the first duplication, one sequence underwent the loss of the TB3 RGD site, as well as several other changes, in order to form fibrillin-1, while the second sequence, coined as fibrillin-2/3, retained the characteristics of the parent sequence and sustained the introduction of a glycine-rich region. The second duplication event coincides with the evolution of tetrapods, thus allowing ray-finned fish to evolve in parallel and develop the present-day form of fibrillin-2/3 found in these species. Following this duplication, one fibrillin copy maintained the features of the parent sequence to become fibrillin-2, while the other, underwent significant evolutionary changes to form fibrillin-3. The transition of the initial tetrapod fibrillin-3 sequence to the fibrillin-3 sequence found in humans and other mammals includes the loss of the RGD site in TB3, the novel introduction of an RGD site in cbEGF18, as well as a reduction of the glycine∶proline ratio in the unique region. Divergence of the fibrillin-3 gene is observed with the evolution of mammals, evident through the loss of the RGD site in TB3 of monotremes and marsupials. Highly conserved sequences throughout the evolution of fibrillins include the pro-protein furin-type processing sites within the unique N- and C-terminal domains, indicating an evolutionarily conserved microfibril assembly mechanism with regards to pro-protein processing. Other notable highly conserved regions in all analyzed fibrillins are the cysteine patterns in the unique N- and C-terminal domains. The results from this study were used to propose key events for the evolution of the three human fibrillins (Fig. 7).

## Supporting Information

Text S1 Additional information about naming fibrillins, zebrafish fibrillins, and sea urchin fibrillin.
(PDF)

Table S1 Sequences analyzed in this study.
(PDF)

Table S2 Additional RGD integrin-binding sites in ancestral fibrillin sequences.
(PDF)
