# Supplementary material for: The Evolution of Extracellular Fibrillins and Their Functional Domains
Source: PLoS One. 2012 Mar 16;7(3):e33560. doi: 10.1371/journal.pone.0033560 (PMC3306419; doi:10.1371/journal.pone.0033560)
Supplement: Text S1 — Additional information about naming fibrillins, zebrafish fibrillins, and sea urchin fibrillin. (PDF) [file pone.0033560.s001.pdf]

## Supplemental Text S1

### General Issues of Naming Fibrillin Sequences

Overall, sequence names as indicated in their respective database entries matched percent identity queries conducted through FASTA comparisons. Furthermore, features unique to each of the three fibrillins, including the location of RGD sites and the differential unique regions were used to distinguish amongst the three genes and proteins. However, conflicting annotation was encountered with certain sequences when these homology criteria were compared with the results of the phylogenetic analysis. A group of sequences, more closely resembling the fibrillin-2 sequence with respect to percent identity and the location of integrin-binding sites in most cases, were grouped within the fibrillin-3 clade, for all trees generated in the analysis, with the exception of the TB7 tree. Species with a fibrillin sequence in this problematic grouping include the frog, chicken, zebra finch, lizard, platypus, opossum, as well as all surveyed ray-finned fish species. In naming sequences, it was not possible to exclusively rely on percent identity and location of RGD sites. As an example, the chicken contains three fibrillin genes; the first has highest homology with human fibrillin-1, while the remaining two sequences both have highest homology, in terms of both percent identity and location of RGD sites, with human fibrillin-2. However, one of these sequences shares 91% identity with human fibrillin-2, while the other shares only 79% identity with human fibrillin-2, and is always placed within the fibrillin-3 clade in the phylogenetic analysis. The latter of these two sequences consistently clusters with the second fibrillin sequence found in the platypus and all ray-finned fish species, as well as with the third fibrillin sequence found in zebra finch, lizard, frog and opossum. Considering these aspects and despite the grouping in the fibrillin-3 clade, we name this sequence as fibrillin-2/3 in all ray-finned fish species, given its divergence from the fibrillin gene that underwent a duplication to form present-day fibrillin-2 and fibrillin-3. In the remaining organisms, all of which contain a total of three fibrillin genes, the problematic sequence is referred to as fibrillin-3, as it

represents an initial form of the gene that had not yet undergone several major evolutionary changes.

## Zebrafish Fibrillins

In zebrafish, a third fibrillin sequence was found and previously annotated as fibrillin-4 by Gansner *et al.* 2008, while other ray-finned fish only contain two fibrillin genes. Zebrafish fibrillin-4 is characterized by its novel proline-glutamine rich region, as well as by a lack of integrin-binding RGD sites (Gansner *et al*. 2008). In order to remain consistent with the sequence annotation used in the present analysis, the sequence referred to as zebrafish fibrillin-2 by Gansner *et al.* is referred to as fibrillin-2/3 in the present manuscript for the reasons previously discussed. In the fibrillin-4 sequence, KGD is in place of the integrin-binding site in TB3, while in TB4, there is a significant lack of homology at the integrin-binding site region. Several possibilities exist to explain the presence of this third fibrillin gene; one possibility is that this gene is in fact the zebrafish fibrillin-2 gene, while a second possibility is that fibrillin-4 is the result of a species-specific duplication of fibrillin-2/3. The distance-based phylogenetic trees generated from TB4, TB6, and TB7, as well as from the concatenated TB domains, place zebrafish fibrillin-4 outside of the fibrillin-2 clade. However this placement is supported by relatively low bootstrap values of 16.1%, 34.2%, 39.4% and 57.7%, respectively. Furthermore, the maximum likelihood phylogenetic reconstruction of concatenated TB domains strongly supports this placement (bootstrap value of 91%). Trees generated from TB1, TB2, and TB3 place this gene outside both the fibrillin-2 and fibrillin-3 clades with higher bootstrap values of 79.1%, 92.2% and 57.8%, respectively. Therefore, it is possible that zebrafish fibrillin-4 is in fact an early version of fibrillin-2, which underwent significant evolutionary changes, including the loss of both RGD sites and changes in amino acid occurrence within the unique region. However, the lack of a third fibrillin in other ray-finned fish renders this hypothesis less likely, unless an actinopterygian-specific loss of the fibrillin-2 gene occurred after the evolution of

zebrafish. With regards to the second possibility, if fibrillin-4 is in fact a species-specific duplication of the fibrillin-2/3 gene, we would anticipate higher homology between zebrafish fibrillin-4 and fibrillin-2/3 when compared to human fibrillin-2. FASTA comparisons reveal that fibrillin-4 has 68% identity with human fibrillin-2 and 67% identity with zebrafish fibrillin-2/3. Human fibrillin-2 and zebrafish fibrillin-2/3 share 75% identity with each other. Gansner *et al.* conclude that fibrillin-4 is not a paralogue created by a genome duplication event of fibrillin-2/3, but state that it does appear to be a distant relative of human fibrillin-2 (Gansner et al. 2008). As such, we believe that the origin of fibrillin-4 likely stems from the zebrafish fibrillin-2/3 gene, however whether it represents an initial form of the fibrillin-2 gene or a species-specific duplication of fibrillin-2/3 remains undetermined.

### Sea Urchin Fibrillin

A report by Whittaker *et al.* claims that sea urchin contains two fibrillin sequences, annotated as Sp-Fibrillin A and Sp-Fibrillin B (Whittaker *et al.* 2006). Contrarily, the data presented here suggests that this organism only contains one *bona fide* fibrillin gene. The Sp-Fibrillin A gene is identical to the single sea urchin fibrillin sequence collected in our study and contains the complete fibrillin sequence signature. The domain signature of the Sp-Fibrillin B sequence was found to consist of the following tandemly arranged domains: cbEGF-TB-cbEGF-like-cbEGF(X4)-TB-cbEGF-like(X6)-cbEGF(x5)-cbEGF-like-cbEGF(X2)-cbEGF-like(X2)-cbEGF-TB. This does not match the fibrillin domain signature which is conserved throughout evolution from jellyfish to humans. Therefore, we did not include this sequence in our analysis.

A recent study by Robertson and coworkers reported that the sea urchin fibrillin sequence contains an extra TB domain followed by four consecutive cbEGF domains near its C-terminus (Robertson *et al*. 2011). Detailed inspection of this sequence reveals an in-frame duplication of five domains: cbEGF28, cbEGF29, cbEGF31, TB6 and cbEGF32 (239 amino acid residues).

While the amino acid sequence of this insert is 100% identical to the duplicated domains, analysis of the nucleotide sequence reveals only 96.1% identity. Of the 28 silent nucleotide changes that were found, 25 occurred in the third position of any given codon, while the remaining three changes occurred within the same codon, all specifying a serine residue. The presence of these nucleotide changes argues against a sequencing or computational error during the assembly of the nucleotide sequence. The complete conservation of these five duplicated domains on the amino acid level indicates a relatively recent event. The sea urchin is the only organism to show such a deviation from the fibrillin signature.

## References


Gansner JM, Madsen EC, Mecham RP, Gitlin JD (2008) Essential role for fibrillin-2 in zebrafish notochord and vascular morphogenesis. Dev Dyn 237: 2844-2861.

Robertson I, Jensen S, Handford P (2011) TB domain proteins: evolutionary insights into the multifaceted roles of fibrillins and LTBPs. Biochem J 433: 263-276.

Whittaker CA, Bergeron KF, Whittle J, Brandhorst BP, Burke RD, et al. (2006) The echinoderm adhesome. Dev Biol 300: 252-266.
