# Supplementary material for: The Evolution of Extracellular Fibrillins and Their Functional Domains
Source: PLoS One. 2012 Mar 16;7(3):e33560. doi: 10.1371/journal.pone.0033560 (PMC3306419; doi:10.1371/journal.pone.0033560)
Supplement: Table S1 — Sequences analyzed in this study. (PDF) [file pone.0033560.s002.pdf]

Table S1 – Sequences analyzed in this study

Species	Common Name	Phylum / Class	Fibrillin Isoform	Accession Number	Database	Modified[a)]
Homo sapiens	Human	Chordata / Mammalia	FBN-1	NM_000138.4	3	No
			FBN-2	NM_001999.3	3	No
			FBN-3	NM_032447.3	3	No
Pan troglodytes	Common chimpanzee	Chordata / Mammalia	FBN-1	XM_001149266.1	3	No
			FBN-2	XM_526999.2	3	No
			FBN-3	ENSPTRT00000019141	2	No
Macaca mulatta	Rhesus macaque	Chordata / Mammalia	FBN-1	XM_001113107.1	3	No
			FBN-2	XR_012189.1	3	Yes
			FBN-3	XM_001097964.2	3	Yes
Otolemur garnettii	Bush baby	Chordata / Mammalia	FBN-1	ENSOGAT00000009590	2	No
			FBN-2	ENSOGAG00000008602	2	No
			FBN-3	ENSOGAG00000012883	2	No
Sus scrofa	Wild pig	Chordata / Mammalia	FBN-1	NM_001001771.1	3	No
			FBN-2	ENSSSCT00000015577	2	Yes
			FBN-3	ENSSSCT00000014847	2	Yes
Bos Taurus	Cow	Chordata / Mammalia	FBN-1	NM_174053.1	3	No
			FBN-2	XM_002688974.1	3	No
			FBN-3	XM_001254849.2	3	Yes
Equus caballus	Horse	Chordata / Mammalia	FBN-1	XM_001502259.2	3	No
			FBN-2	XM_001918259.1	3	No
			FBN-3	XM_001916998.1	3	Yes
Canis lupus familiaris	Dog	Chordata / Mammalia	FBN-1	XM_535468.2	3	No
			FBN-2	XM_538612.2	2	No
			FBN-3	XM_542124.2	3	No
Ailuropoda melanoleuca	Giant panda	Chordata / Mammalia	FBN-1	EFB29915.1	3	No
			FBN-2	GL192553.1	3	No
			FBN-3	EFB25575.1	3	No
Dasypus novemcinctus	Nine-banded armadillo	Chordata / Mammalia	FBN-1	ENSDNOT00000007233	2	No
			FBN-2	ENSDNOT00000015787	2	No

Cavia porcellus	Guinea pig	Chordata / Mammalia	FBN-1	Q924N1_CAVPO	2	Yes
			FBN-2	ENSCPOT00000000731	2	Yes
			FBN-3	ENSCPOT00000027611	2	Yes
Rattus norvegicus	Brown rat	Chordata / Mammalia	FBN-1	AF135059.1	3	No
			FBN-2	AF135060.1	3	No
Mus musculus	House mouse	Chordata / Mammalia	FBN-1	NM_007993.2	3	No
			FBN-2	NM_010181.2	3	No
Monodelphis domestica	Gray short-tailed opossum	Chordata / Mammalia	FBN-1	XM_001369811.1	3	No
			FBN-2	ENSMODT00000001530	2	No
			FBN-3[b)]	XM_001376665.1	3	Yes
Ornithorhynchus anatinus	Platypus	Chordata / Mammalia	FBN-1	Ultra 375 scaffold, contigs 215463.1, 187161.1, 73890.2	3, 6	Yes
			FBN-3[b)]	ENSOANT00000010193	2	Yes
Taeniopygia guttata	Zebra finch	Chordata / Aves	FBN-1	XM_002196584.	3	Yes
			FBN-2	XM_002189166.1	3	No
			FBN-3[b)]	ENSTGUT00000001010	2	No
Gallus gallus	Chicken	Chordata / Aves	FBN-1	XM_413815.2	3	Yes
			FBN-2	XM_424715.2	3	Yes
			FBN-3[b)]	XM_418173.2	3	No
Anolis carolinensis	Green anole lizard	Chordata / Reptilia	FBN-1	ENSACAT00000005310	2	Yes
			FBN-2	ENSACAT00000002353	2	Yes
			FBN-3[b)]	ENSACAT00000014803	2	Yes
Xenopus tropicalis	Western clawed frog	Chordata / Amphibia	FBN-1	genomic scaffold 221	1, 6	Yes
			FBN-2	462390	1	Yes
			FBN-3[b)]	ESTs	5, 6	Yes
Takifugu rubripes	Japanese pufferfish	Chordata / Actinopterygii	FBN-1	ENSTRUT00000043497	2	Yes
			FBN-2/3[b)]	ENSTRUT00000033093	2	Yes
Tetraodon nigroviridis	Spotted green pufferfish	Chordata / Actinopterygii	FBN-1	ENSTNIT00000002027	2	Yes
			FBN-2/3[b)]	ENSTNIT00000001247	2	Yes
Oryzias latipes	Japanese medaka fish	Chordata / Actinopterygii	FBN-1	ENSORLG00000002614	2	Yes
			FBN-2/3[b)]	ENSORLT00000006744	2	Yes

Danio rerio	Zebrafish	Chordata / Actinopterygii	FBN-1	ENSDARP00000058536	2	Yes
			FBN-2/3[b)]	NM_001135790.1	3	No
			FBN-4	XM_681408.4, XM_686017.4, EU854565.1	3	No
Branchiostoma floridae	Amphioxus	Chordata / Leptocardii	FBN	127734	1	No
Petromyzon marinus	Sea lamprey	Chordata / Cephalaspidomorphi	FBN	contig 1915	5, 6	Yes
Ciona intestinalis	Sea squirt	Chordata / Ascidiacea	FBN	XM_002119952.1	3	Yes
Ixodes scapularis	Blacklegged tick	Arthropoda / Arachnida	FBN	XM_002412312.1	3	No
Apis mellifera	European honey bee	Arthropoda / Insecta	FBN	Group 11 scaffold	4, 6	Yes
Harpegnathos saltator	Jerdon's jumping ant	Arthropoda / Insecta	FBN	EFN77739	3	No
Tribolium castaneum	Red Flour Beetle	Arthropoda / Insecta	FBN	XM_969251.2	3	Yes
Pediculus humanus corporis	Human body louse	Arthropoda / Insecta	FBN	XM_002428733.1	3	No
Acyrthosiphon pisum	Pea aphid	Arthropoda / Insecta	FBN	XM_001945936.1	3	Yes
Daphnia pulex	Water flea	Arthropoda / Branchiopoda	FBN	315775	1	No
Lottia gigantean	Sea snail	Mollusca / Gastropoda	FBN	232356	1	Yes
Capitella teleta	Worm	Annelida / Polychaeta	FBN	223950	1	Yes
Strongylocentrotus purpuratus	California purple sea urchin	Echinodermata / Echinoidea	FBN[b)]	XM_782436.2	3	Yes
Hydra magnipapillata	Hydra	Cnidaria / Hydrozoa	FBN	XR_053678.1	3	Yes
Podocoryne carnea	Jellyfish	Cnidaria / Hydrozoa	FBN	L39930.1	3	Yes
Nematostella vectensis	Starlet sea anemone	Cnidaria / Anthozoa	FBN	genomic scaffold 113	3, 6	Yes

[1] DOE Joint Genome Institute (http://www.jgi.doe.gov)
[2] Ensembl (http://uswest.ensembl.org)
[3] NCBI Nucleotide Database (Nuccore) (http://www.ncbi.nlm.nih.gov/nuccore)
[4] UCSC Genome Bioinformatics (http://genome.ucsc.edu)
[5] EMBL European Bioinformatics Institute (http://www.ebi.ac.uk)
[6] These sequences were obtained by manual sequence reconstruction from various genomic scaffolds and contigs
[a)] Indicated sequences have been modified to remove missense regions and add missing regions
[b)] These sequence names are in conflict between our analysis and annotated fibrillin database entries; see supplemental text for further information
