# Supplementary material for: The Evolution of Extracellular Fibrillins and Their Functional Domains
Source: PLoS One. 2012 Mar 16;7(3):e33560. doi: 10.1371/journal.pone.0033560 (PMC3306419; doi:10.1371/journal.pone.0033560)
Supplement: Table S2 — Additional RGD integrin-binding sites in ancestral fibrillin sequences. (PDF) [file pone.0033560.s003.pdf]

Table S2 – Additional RGD integrin-binding sites in ancestral fibrillin sequences

Species	Domain	Comments
Harpegnathos saltator	EGF1	Honey bee has RGE
Acyrthosiphon pisum	cbEGF2	
Ciona intestinalis	cbEGF8	
Harpegnathos saltator<br>*Apis mellifera*<br>*Acyrthosiphon pisum*	cbEGF18	This sequence is further downstream of the fibrillin-3 RGD site found in cbEGF18 in humans and chimpanzees; other sequences have XGD
Branchiostoma floridae	cbEGF28	
Lottia gigantea	cbEGF30	
Pediculus humanus corporis<br>*Acyrthosiphon pisum*	TB6	Similar non-RGD sequences in other arthropods, sea lamprey, lizard, frog, chicken, and zebrafish
Nematostella vectensis<br>*Ixodes scapularis*	cbEGF35	
